# De Novo Enzyme Design Using Rosetta3

**DOI:** 10.1371/journal.pone.0019230

**Published:** 2011-05-16

**Authors:** Florian Richter, Andrew Leaver-Fay, Sagar D. Khare, Sinisa Bjelic, David Baker

**Affiliations:** 1 Department of Biochemistry, University of Washington, Seattle, Washington, United States of America; 2 Interdisciplinary Program in Biomolecular Structure and Design, University of Washington, Seattle, Washington, United States of America; 3 Department of Biochemistry, University of North Carolina, Chapel Hill, North Carolina, United States of America; University of South Florida College of Medicine, United States of America

## Abstract

The Rosetta de novo enzyme design protocol has been used to design enzyme catalysts for a variety of chemical reactions, and in principle can be applied to any arbitrary chemical reaction of interest, The process has four stages: 1) choice of a catalytic mechanism and corresponding minimal model active site, 2) identification of sites in a set of scaffold proteins where this minimal active site can be realized, 3) optimization of the identities of the surrounding residues for stabilizing interactions with the transition state and primary catalytic residues, and 4) evaluation and ranking the resulting designed sequences. Stages two through four of this process can be carried out with the Rosetta package, while stage one needs to be done externally. Here, we demonstrate how to carry out the Rosetta enzyme design protocol from start to end in detail using for illustration the triosephosphate isomerase reaction.

## Introduction

There has been exciting recent progress in computational enzyme design. Active enzymes have been designed for a variety of reactions including the Diels-Alder reaction [1], the Kemp elimination [2], and the retro-aldol reaction [3]. This paper describes in detail the protocol used to design these catalysts to help researchers apply the method to new reactions, and discusses routes for further improvement of the methods.

**Figure 1:**
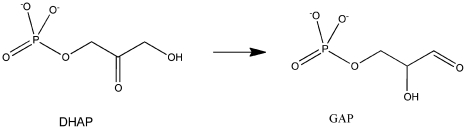
Isomerization of DiDihydroxy-acetone-phosphate (DHAP) to Glyceraldehyde-3-phosphate (GAP).

**Figure 2:**
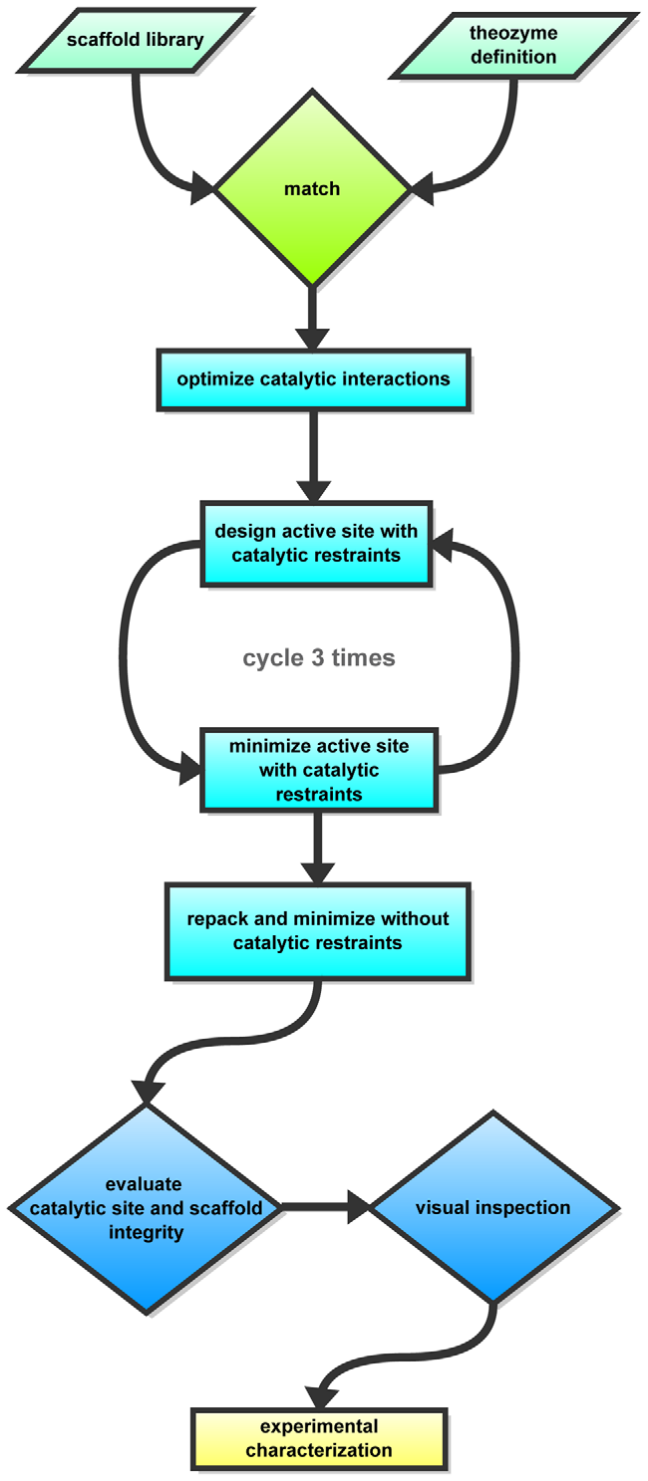
Flowchart of the enzyme design protocol, colored according to the different stages. Stage 1: light green; Stage 2: green; Stage 3: cyan; Stage 4: blue.

Enzymes accelerate reactions through interactions that stabilize the transition state [4]. The Rosetta enzyme design protocol starts with minimalist active site descriptions consisting of transition state models surrounded by disembodied side chain and backbone functional groups positioned optimally for catalysis, so called theozymes [5]. The Rosetta “matching” algorithm [6] is then used to identify constellations of backbone positions in a set of scaffold proteins where the minimalist active sites can be realized. For each such recapitulation of the minimalist active site in a protein scaffold, the Rosetta design methodology is used to optimize the surrounding residues for transition state binding affinity and catalysis. Here, the Rosetta enzyme design protocol, which has been recently re-implemented as part of the re-architechturing of the Rosetta molecular modeling program [7], is described in detail using as an example the isomerization of Dihydroxy-acetone-phosphate (DHAP) to Glyceraldehyde-3-phosphate (GAP) [8](Figure 1). Figure 2 shows an overview over the different stages of the design process.

## Methods

### 1) Choice of catalytic mechanism and corresponding theozyme

The first step in the enzyme design process is to model the reaction transition state(s) and decide on a set of idealized active site descriptions consisting of disembodied side chain and backbone functional groups surrounding the transition state in positions optimal for catalysts. The latter include for example hydrogen bond acceptors and donors for proton abstraction and proton donation, positive charges to stabilize developing negative charge in the transition state, etc. These active site descriptions and geometries can be obtained from quantum chemistry saddle point calculations, as in the case of the theozymes pioneered by Ken Houk's group [9], from analogy to active sites of known enzymes, from chemical intuition, or in practice a combination of the above.

Once a theozyme is defined, it needs to be expressed in terms of a Rosetta geometric constraint file, a “cstfile.” The information in this cstfile is used both by the Rosetta matcher to try to graft the desired theozyme onto a scaffold structure, and by the enzyme_design code to restrain the theozyme residues to the desired theozyme geometry during sequence optimization and gradient-based minimization.

The cstfile consists of blocks; for each interaction between two residues, (i.e. for each theozyme interaction), there needs to be one block. The example below describes the interaction between a Glu or an Asp and a ligand abbreviated with name 1n1. In this cstfile, there needs to be a block of the following format for each catalytic interaction:

CST::BEGIN

TEMPLATE:: ATOM_MAP: 1 atom_name: C1 C2 O2

TEMPLATE:: ATOM_MAP: 1 residue3: 1n1

TEMPLATE:: ATOM_MAP: 2 atom_type: OOC,

TEMPLATE:: ATOM_MAP: 2 residue1: ED

CONSTRAINT:: distanceAB: 3.06 0.2 100. 0 0

CONSTRAINT:: angle_A: 73.60 10.0 80.0 360. 1

CONSTRAINT:: angle_B: 120.00 15.0 80.0 360. 1

CONSTRAINT:: torsion_A: -101.20 15.0 60.0 360. 1

CONSTRAINT:: torsion_AB: 180.00 90.0 0.00 360. 3

CONSTRAINT:: torsion_B: 180.00 15.0 0.00 360. 1

CST::END

The information in this block defines constraints between three atoms on residue 1 and three atoms on residue 2. Up to six parameters can be specified, representing the ligand's six rigid-body degrees of freedom. These parameters are given as one distance, two angles, and three dihedrals.

The Records indicate the following:

‘CST::BEGIN’ and ‘CST::END’ indicate the beginning and end of the respective definition block for this catalytic interaction. The ‘TEMPLATE:: ATOM_MAP:’ records indicate what atoms are constrained and what type of residue they are in. The number in column 3 of these records indicates which catalytic residue the record relates to. It has to be either 1 or 2.

**Figure 3:**
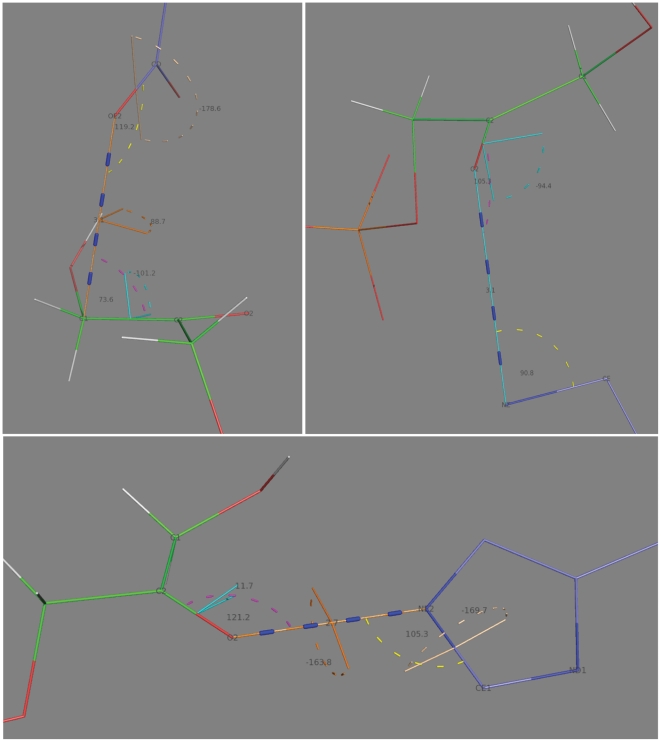
Theozyme geometries. Top left: Interaction 1; top right: Interaction 3; bottom: Interaction 2; Color scheme: distanceAB: blue, angle_A: purple, angle_B: yellow, torsion_A: cyan, torsion_AB: orange, torsion_B: ligt brown.

The ‘atom_name’ tag specifies exactly which 3 atoms of the residue are to be constrained. It has to be followed by the names of three atoms that are part of the catalytic residue or ligand. In the above example, for catalytic residue 1, the ligand, atom 1 is C1, atom 2 is C2, and atom3 is O2. The geometry specified is visualized in Figure 3, top left panel.

The ‘atom_type’ tag is an alternative to the ‘atom_name’ tag. It allows more flexible definition of the constrained atoms. It has to be followed by the Rosetta atom type of the residue's atom 1. In case this tag is used, Rosetta will set atom 2 as the `̀base atom" (the parent for an atom in the tree rooted at the backbone growing out along the side chain) of atom 1, and will set atom 3 as the base atom for atom 2. There are two advantages to using the ‘atom_type’ tag: first, it allows constraining different residue types with the same file, e.g. if a catalytic hydrogen bond is required, but either a SER or THR would do. Second, if a catalytic residue contains more than one atom of the same type (e.g. atoms OD1 and OD2 of Asp), but it doesn't matter which of these atoms mediates the constrained interaction, using this tag will cause Rosetta to evaluate the constraint for all of these atoms separately and pick the one with lowest score, i.e. the ambiguity of the constraint will automatically be resolved.

The ‘residue1’ or ‘residue3’ tag specifies what type of residue is constrained. ‘residue3’ needs to be followed by the name of the residue in 3-letter abbreviation. ‘residue1’ needs to be followed by the name of the residue in 1-letter abbreviation. As a convenience, if several similar residue types can fulfill the constraint (e.g. either Asp or Glu), the ‘residue1’ tag can be followed by a string of 1-letter codes of the allowed residues ( e.g. “ED” for Asp and Glu, or “ST” for Ser and Thr ).

The ‘CONSTRAINT::’ records specify the parameters and the strengths of the constraint applied between the two residues. Each of these records is followed by one string and four numbers. The string can have the following allowed values:

‘distanceAB’ means the distance Res1:Atom1  = Res2:Atom1, i.e. the distance between atom1 of residue 1 and atom1 of residue 2.

‘angle_A’ is the angle Res1:Atom2 - Res1:Atom1 - Res2:Atom1

‘angle_B’ is the angle Res1:Atom1 - Res2:Atom1 - Res2:Atom2

‘torsion_A’ is the dihedral Res1:Atom3 - Res1:Atom2 - Res1:Atom1 - Res2:Atom1

‘torsion_AB’ is the dihedral Res1:Atom2 - Res1:Atom1 - Res2:Atom1 - Res2:Atom2

‘torsion_B’ is the dihedral Res1:Atom1 - Res2:Atom1 - Res2:Atom2 - Res2:Atom3

Each of these strings is followed by 4 (optionally 5 ) columns of numbers: x0, xtol, k, covalent/periodicity, and number of samples. The 1st column, x0, specifies the optimum distance x0 for the respective value. The 2nd, xtol, column specifies the allowed tolerance xtol of the value. The 3rd column specifies the force constant k, or the strength of this particular parameter. If x is the value of the constrained parameter, the score penalty p(x) applied will be:



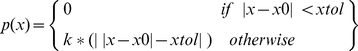



The 4th column has a special meaning in case of the distanceAB parameter. It specifies whether the constrained interaction is covalent or not. 1 means covalent, 0 means non-covalent. If the constraint is specified as covalent, Rosetta will not evaluate the vdW term between Res1:Atom1 and Res2:Atom1 and their [1], [3] neighbors.

For the other 5 parameters, the 4th column specifies the periodicity per of the constraint. For example, if x0 is 120 and per is 360, the constraint function will have a its minimum at 120 degrees. If x0 is 120 and per is 180, the constraint function will have two minima, one at 120 degrees and one at 300 degrees. If x0 is 120 and per is 120, the constraint function will have 3 minima, at 120, 240, and 360 degrees.

The 5th column is optional and specifies how many samples the matcher, if using the classic matching algorithm, will place between the x0 and x0+- tol value. The matcher interprets the value in this column as the number of sampling points between x0+ xtol and x0– xtol, i.e. in the above example, for angle_A, the matcher will sample values 63.6, 73.6, and 83.6 degrees. Generally, if the value in this column is n, the matcher will sample 2n+1 points for the respective parameter. Note that the number of samples is also influenced by the periodicity, since the matcher will sample around every x0.

When determining how many different values to sample for each parameter, it is important to remember that the number of different ligand placements attempted for every protein rotamer built is equal to the product of the samples for each of the 6 parameters. For example, in the above block there is one sample for distanceAB, 3 samples for angle_A, 3 samples for angle_B, 3 samples for torsion_A, 3 samples for torsion_B, and 7 samples for torsion_AB, meaning that for every protein rotamer, the matcher will attempt to place the ligand in a total of 1*3*3*3*3*7  = 567 different conformations.

### 2) Matching: identifying sites in the scaffold library where the theozyme can be placed

In this stage, the hypothetical theozyme will be placed into an existing protein structure with the help of the RosettaMatch module. The inputs for this stage are the theozyme expressed in the cstfile format as described above, and a list of protein scaffolds. The RosettaMatch executable will be run once for each scaffold and, if the theozyme and the respective scaffold are compatible, will output a number of so-called matches. A match is defined as the theozyme grafted into a scaffold, i.e. the amino acid side chains of the theozyme have been placed on the scaffold backbone, the ligand has been placed into a cavity of the scaffold without clashing with the protein backbone or the theozyme side chains, and the geometric relation between the ligand and the theozyme side chains is as specified in the theozyme.

To run RosettaMatch, the user has to prepare each scaffold and decide which of two available algorithms to use for each side chain of the theozyme. Preparing the scaffold consists of deciding which residues of the scaffold should be considered when trying to place the theozyme.

#### Preparing the scaffold for matching

Usually only a subset of the scaffold residues are considered during the matching process, for two reasons: first, residues lining a concave pocket or cleft of the scaffold are more likely to form a binding site than residues that are buried in the hydrophobic core or residues that protrude into the solvent. Trying to design a binding site in the core of the protein is problematic because it will likely have a negative effect on the stability of the protein, while creating a binding site on the surface of the protein is difficult because there are possibly not enough side chains to contact the ligand from several sides and thus form a binding surface that is complementary in shape to the ligand. Second, the more residues that are considered by the matcher, the higher the computational requirements in terms of runtime and memory become, and for theozymes with many degrees of freedom the matching runtime can quickly become the bottleneck of the whole process.

Therefore, for each scaffold that is considered by the matcher, a subset of residues most likely to be part of the binding site need to be selected, such as residues lining a pocket or cleft. In case the scaffold was crystallized with a natural ligand, one could for example select all residues that are within a certain distance from that natural ligand.

#### Choosing a matching algorithm for each theozyme interaction

RosettaMatch can place the theozyme side chains through two algorithms: classic matching as introduced by Zanghellini et al. [6], and "secondary matching." Both algorithms can be used in the same matching run.

Classic matching has been described in detail before [6]. Briefly, for every interaction/side chain of the theozyme, the matcher builds rotamers at each of the scaffold active-site positions. For each rotamer built, the ligand is placed according to the geometry specified in the theozyme/cstfile. To use the classic matching algorithm, sample values must be given for all 6 parameters connecting the side chain to the transition state. Note that depending on the theozyme and the set of sample values specified in the cstfile, the number of different ligand placements for each side chain rotamer can grow to be quite large. Each ligand placement is checked for collisions with the scaffold's backbone, and, if there are none, the location and orientation of the ligand in space is recorded in a 6D coordinate (3 Euclidean coordinates and 3 Eulerian coordinates). The 6D coordinate for each collision free ligand placement along with the side chain rotamer used to build this coordinate, is called a "hit."

For a theozyme interaction/side chain to be placed with the secondary matching algorithm, the matcher proceeds as follows: First, just like in classic matching, rotamers at all scaffold active-site positions are built. Then, for each rotamer r, the geometry between r and each of the previously generated hits is evaluated. If the geometry of rotamer r and a particular hit h is compatible with the desired theozyme geometry, the 6D coordinate of the ligand is copied from h and stored with rotamer r as a new hit for this theozyme interaction.

After hits have been generated for all N side chains/interactions of the theozyme, all hits are then binned according to the 6D coordinate of the ligand and placed into a hash table. Then, the hash bins are checked for whether they contain at least one hit from each of the N theozyme side chains. For every bin that does, the ligand placements stored in it, together with the side chain rotamers they were built from, are considered a "match", i.e. a successful graft of the theozyme onto the scaffold.

Classic matching and secondary matching each have their own advantages and disadvantages. Which algorithm to choose for a given theozyme interaction depends on several factors.

There are two advantages to the secondary matching algorithm: first, since the ligand is not built from the side chain rotamer, but instead taken from a previously generated hit, not all six degrees of freedom must be specified. For example, if a theozyme interaction depends only the distance and the two angles, while the three dihedrals are unimportant, then the secondary matching algorithm can be given ranges for only the important parameters, while ignoring the unimportant parameters. If such an interaction were to be described to the classic matching algorithm, the unimportant dihedral parameters would have to be sampled over the whole 360 degree range, resulting in long running times. Sampling these dihedrals coarsely at 10 degree increments still requires building 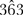
∼46 thousand ligand conformations per assignment to the other 3 parameters per side chain rotamer.

Second, the secondary matching algorithm can also be used to find theozyme interactions between two side chains. Since both the ligand placement and the rotamer from which it stems are stored in a hit, the secondary matching algorithm can equally well evaluate the geometry between a rotamer built for one theozyme interaction and a rotamer for a previously generated hit.

The advantage of the classic matching algorithm is that it performs considerably faster than the secondary match algorithm in cases where a large number of ligand placements have been generated for preceding interactions of the theozyme. The speed of placing one theozyme interaction with the secondary matching algorithm decreases with an increasing number of previous ligand placements, since every one of them needs to be examined again. The classic match algorithm, on the other hand, always generates the ligand placements from the coordinates of the side chain rotamer and the information in the geometric constraint file, and is thus independent of previously generated ligand placements. It also is less sensitive to the lever-arm effect than secondary matching; secondary matching may not capture ligand interaction geometries very accurately forcing the user either to tolerate very broad ranges of values for a particular interaction or to sample ligand geometries very densely in other theozyme interactions.

As a rule of thumb, secondary matching should be used for theozyme interactions where not all six degrees of freedom are clearly defined and for side chain-side chain theozyme interactions, while classic matching should be used in other cases. Note that since secondary matching must rely on the hits generated prior to its execution, classic matching must be used for the first theozyme interaction.

Command line options affecting this stage:

Matching is carried out by the match executable available in the Rosetta 3.2 release and is sensitive to the following command line options:

-extra_res_fa <filename>      path to rosetta parameter file of ligand to be matched

-lig_name <string>        name of the ligand to be matched

-geometric_constraint_file <filename> path to constraint file / theozyme

-s <filename>            path to scaffold pdb

-scaffold_active_site_residues <filename>file containing what residues of the scaffold to match at

-ex1, -ex2 <value>         optional parameter governing size of rotamer library

### 3) Designing the found sites

After matches have been found, optimal residue identities for other scaffold positions need to be determined to build an active site that is complimentary in shape to the ligand while also stabilizing the catalytic side chains in their matched conformations. The basic Rosetta enzyme design protocol used for this consists of four steps, and is all carried out by the enzyme_design executable available in the Rosetta 3.2 package:

- Determining which residues to design and which to repack
- Optimizing the catalytic interactions
- Cycles of sequence design/minimization (with catalytic constraints if specified)
- Unrestrained fixed sequence rotamer pack /minimization

#### Determining which residues to design and which to repack

There are two ways of doing this: using a standard Rosetta resfile to exactly specify which residues are allowed at which position or automatic detection of the design region. In case there is only a small number of different starting structures, it is probably better to invest the time and use intuition to decide which positions in the protein to redesign or repack and which amino acids to allow.

In case there are a lot of input structures to be designed, it is also possible to automatically determine which residues to redesign.

Rosetta can divide the protein's residues into 5 groups of increasing distance from the ligand:

1. Residues that have their Cα within a distance cut1 Å of any ligand heavy atom will be set to designable
2. Residues that have their Cα within a distance cut2 of any ligand heavy atom and the Cβ closer to that ligand atom than the Cα will be set to designable. cut2 has to be larger than cut1
3. Residues that have their Cα within a certain distance cut3 of any ligand heavy atom will be set to repackable. cut3 has to be larger than cut2
4. Residues that have their Cα within a distance cut4 of any ligand heavy atom and the Cβ closer to that ligand atom will be set to repackable. cut4 has to be larger than cut3
5. All residues not in any of the above 4 groups are kept static.

Residues declared as catalytic in the input pdb will always be repackable (except if turned off by an option). At residue positions that are set to designable, every amino acid except cysteine will be allowed. Values (in Å) for the different cuts commonly used are: 6.0 (cut1), 8.0 (cut2), 10.0 (cut3), and 12.0 (cut4).

Command line options affecting this stage:

-resfile \<name of resfile\>   specifies the use of a resfile

-enzdes:detect_design_interface invokes automatic detection of designable region

-enzdes:cut1 \<value\> value used for cut1 (in Å)

-enzdes:cut2 \<value\> value used for cut2 (in Å)

-enzdes:cut3 \<value\> value used for cut3 (in Å)

-enzdes:cut4 \<value\>   value used for cut4 (in Å)

-enzdes:fix_catalytic_aa prevents catalytic residues from being repacked or minimized

Further, these two ways of declaring the design and repack regions can be combined, i.e. a resfile and the detect_design_interface mechanism can be used concurrently. If the default behavior in the resfile is set to ‘AUTO’, the behavior of every residue which is not specifically declared in the resfile will be determined according to the -detect_design_interface logic.

#### Optimizing catalytic interactions

This stage consists of a gradient-based minimization of the input structure before design. During this minimization, all active site residues that are not catalytic (i.e. not part of the theozyme) are mutated to alanine (i.e. the active site is reduced to substrate and catalytic residues only), and a reduced energy function that does not contain vdW-attractive or solvation terms is used for the minimization. Restraints as specified in the cstfile are placed on the interactions between catalytic residues and the ligand. The purpose of this stage is to move the substrate to a position where the catalytic interactions are as ideal as possible.

Command line options affecting this stage:

-enzdes:cst_opt  will invoke this stage

-enzdes:bb_min   optional but recommended. Allows the backbone to be slightly flexible during the minimization

-enzdes:chi_min  optional but recommended. Allows the dihedrals of the catalytic residues to move during the minimization

For backbone minimization, only the backbone phi/psi angles of residues in the designable/repackable region will be allowed to move. A special fold tree [10] is created to constrain backbone movement to the designed site, i.e. there will be no conformational changes in regions that are neither repackable nor designable. Further, to prevent the backbone of the active site from moving considerably during the gradient-based minimization, the Cα atoms are restrained to within 0.5 Å of their original positions.

An alternative to the gradient-based restraint optimization is running a short docking trajectory of the ligand with Monte Carlo rigid body sampling. It is invoked by

-enzdes:cst_predock    invokes this stage

-enzdes:trans_magnitude largest allowed displacement of the ligand (in Å)

-enzdes:rot_magnitude   largest allowed rotation of the ligand (in degrees)

-enzdes:dock_trials    number of rigid body moves attempted

The trans_magnitude and rot_magnitude values are sampled with a Gaussian distribution of zero mean and one standard deviation. The rotation and translation center is taken as the centroid of the set of ligand atoms which have distance restraints to the protein. This ensures the most efficient sampling of the ligand with respect to the restraints. As in Bi, all designable residues are mutated to alanine to allow the ligand to sample the whole active site region prior to design.

#### Cycles of sequence design and minimization

This is where the actual sequence design happens. At the designable positions, the Rosetta standard Monte Carlo algorithm is employed to find a new lower energy sequence for the non-catalytic residues. The catalytic restraints are kept on through the entirety of this stage. After sequence design, the resulting structure is minimimized. These two steps are typically iterated upon a small number of times (2–4).

Command line options affecting this stage:

-enzdes:cst_design     will invoke this stage

-enzdes:design_min_cycles how many iterations of design/minimization will be done

-enzdes:lig_packer_weight determines the relative importance of protein-substrate interactions vs. protein-protein interactions in the sequence selection calculation

-enzdes:cst_min       necessary to invoke minimization after sequence design

-enzdes:bb_min        same as for stage B

-enzdes:chi_min        same as for stage B

-packing:ex1 optional but highly recommended. improved rotamer sampling around the first dihedral for every amino acid

-packing:ex2 optional but highly recommended. improved rotamer sampling around the second dihedral for every amino acid

-packing:use_input_sc    optional but highly recommended. include the input rotamer of every side chain in the calculation

-packing:soft_rep_design  triggers use of the soft-repulsive force field in design.

-packing:linmem_ig 10    optional but highly recommended. speeds up the sequence design step while at the same time reducing memory requirements.

-packing:unboundrot     optional. pdb files that contain additional rotamers to use in rotamer packing calculations.

#### Unrestrained fixed-sequence rotamer packing and minimization

After Rosetta has designed a new sequence, a final repack/minimization will be done without the catalytic restraints. This is to check whether the designed sequence actually holds the catalytic residues in their catalytically active conformations; in a good design, the catalytic residues should adapt their theozyme conformations without artificial restraints enforcing them.

Command line options affecting this stage:

-enzdes:no_unconstrained_repack will prevent this stage from being invoked

-packing:ex1 same as for stage C

-packing:ex2 same as for stage C

-packing:use_input_sc same as for stage C

-enzdes:cst_min same as for stage C

-enzdes:bb_min same as for stage B+C

-enzdes:chi_min same as for stage B+C

### 4) Evaluating and ranking the resulting designed sequences

In a typical enzyme design project, often hundreds or thousands of input structures will be designed. Typically when matches are found, they produce many structures which are very similar to each other (i.e. they derive from matches with very similar ligand placements). It is also recommended to redesign every starting structure a few times, since the stochastic Monte Carlo algorithm can lead to slightly different results every time. Together, this means that there are thousands of designs produced by stage 3. The problem then becomes analyzing, and ranking all of the produced structures to find the best handful worth experimentally characterizing. There is no perfect or ideal way to do this, and only one of many possibilities is described here.

Every model that is output by Rosetta has the scores broken down by residue and score type appended after the atom records. One can simply select the model that has the best overall score, or the best ligand score, or the best constraint score, etc.

However, the Rosetta scores don't necessarily capture all the important characteristics of a given design. The enzyme_design application is set up to evaluate each output structure with respect to the following additional properties and metrics:

-number of hydrogen bonds (in the whole protein and catalytic residues)

-number of buried unsatisfied polar atoms (whole protein/catalytic residues)

-non-local contacts (i.e. contacts between residues that are far away in sequence, for both whole protein/catalytic residues)

-score across the interface between protein/ligand

-packstat [13] of the designed structure with and without ligand present

if the option -out:file:o <filename> is active, a scorefile containing will be written that contains one line for every output structure. The column labels in the score file have the following meaning:

General syntax:

pm  =  Column labels ending in "_pm" are determined using a pose metric calculator

The catalytic/constrained residues are SR1, SR2, SRN for N residues. e.g. if there is one catalytic residue only SR1. Note that if the same catalytic residue is involved in multiple interactions (such as in a triad), it will appear multiple times.

The ligand is the SR(N+1) and it is the last SR, e.g. if there is one catalytic residue it is SR2.

total_score: score (excluding the restraint score)

fa_rep:    full atom repulsive score

hbond_sc:   hbond side chain score

all_cst:       all restraint score

tot_pstat_pm:    pack statistics [13]

total_nlpstat_pm: pack statistics without the ligand present

tot_burunsat_pm:  buried unsatisfied polar atoms

Command line options affecting this stage:

-out:file:o will trigger writing of the output file

-final_repack_without_ligand will repack the design apo-structure and evaluate RMSD

## Results

To demonstrate the Rosetta enzyme design protocol, a full calculation for the triose phosphate isomerase (TIM) reaction(Figure 1) was carried out. This reaction is an essential component of glycolysis, and is one of the best-studied model reactions in enzymology [8]. High-resolution crystal structures of native enzymes have been obtained [11], and the fold they adopt was named after the reaction [12]. Numerous mechanistic, mutational and computational studies have been performed. Here, we try to design a TIM active site into a thermophilic scaffold of the same fold family as the native TIM.

### 1) Defining the theozyme

**Figure 4:**
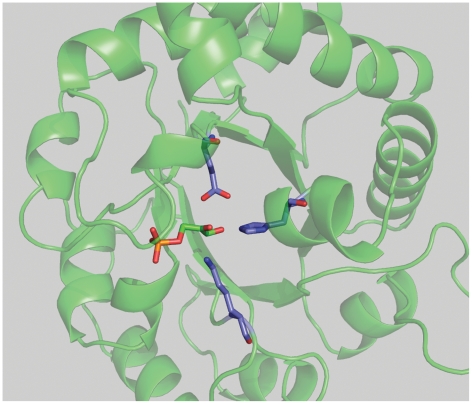
Crystal structure of *S. cerevisiae* TIM with substrate and the three most critical catalytic residues shown in stick representation.

**Figure 5:**
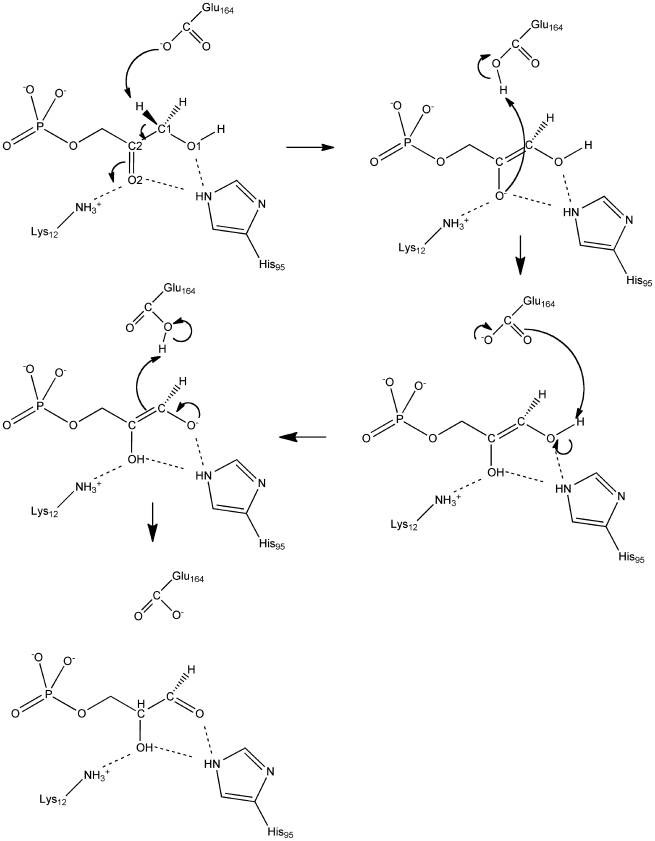
Proposed reaction mechanism of the DHAP to GAP isomerization as catalyzed by *S. cerevisiae* TIM. In the top left panel, substrate atoms are labeled according to their label in the theozyme model.

Defining the theozyme is easy in this case, since several crystal structures of native enzymes exist, and the most important residues in the active site have been determined previously [11]. Quantum mechanical calculations of the natural enzyme have rationalized the geometry observed between the catalytic residues and the substrate[8]. The theozyme is based on the crystal structure of *S.cerevisiae* TIM (PDB code 1ney, Figure 4) and defined according to a mechanism advocated by Guallar *et al.* [8], depicted in Figure 5. It contains three side chains: 1) a Glu/Asp (Glu165 in 1ney) to carry out two proton shuffling steps, 2) a His (His95 in 1ney) to polarize O1 and O2, the two substrate oxygen atoms that change hybridization during the reaction, and 3) a Lys (Lys12 in 1ney) to additionally polarize O2 and facilitate the initial enolate formation.

The geometries used in matching, shown in Figure 3 and summarized in Table S1, are fairly constrained for the first two side chains and more relaxed for the third interaction. For Interaction 1, the carboxylate moiety of an Asp or Glu side chain needs to be in a position to abstract the *pro-R* proton from C1. This dictates the values for distance_AB, angle_A, and torsion_A to be within a fairly small range. Further, since the deprotonation is happening through the empty *anti* sp2 orbital of one carboxylate-O, angle_B and torsion_B are clearly defined. There is more freedom in the last parameter, torsion_AB, which governs the rotation around the DHAP:C1 – Asp:OD1/2/ Glu:OE1/2, and accordingly the matcher is set to sample a wider range for this.

For the second side chain, a His, the protonated N of the imidazole ring needs to be between DHAP:O1 and DHAP:O2, within hydrogen bonding distance to both, and in the plane created by DHAP:C1, DHAP:C2, and DHAP:O2. This requirement clearly determines the values for distance_AB, angle_A, and torsion_A. The protonated N-sp2 orbital is pointing between the two oxygen atoms, and thus determining values for angle_B and torsion_B. There is a little more variability possible for torsion_AB, which governs the relative rotational orientation of the imidazole plane to the substrate plane.

The third interaction, between the Lys and the DHAP, is more loosely defined. The desired hydrogen bonding geometry between Lys:NZ and DHAP:O2, both sp3 hybridized after the initial reaction step, neccessitates tight ranges for the values for distanceAB, angle_A, and angle_B. To restrict the Lys to the side of the DHAP plane opposite of the theozyme Asp/Glu, torsion_A is also restricted. The values for torsion_AB and torsion_B, on the other hand, do not have much influence on the quality of the interaction, and therefore are allowed to have any arbitrary value.

### 2) Matching

In the example presented here, a TIM active site will be placed into a thermophilic scaffold with a TIM β/α barrel fold [12]. Six scaffolds of this fold from three thermophilic organisms, listed in table 1, were selected from the PDB. None of these proteins have been annotated as catalyzing the TIM reaction. For each scaffold, residues lining the natural binding pocket were selected as match positions.

**Table 1:** Matching results.

PDB ID	Natural function	Source organism	Number of matches	runtime/seconds
1tml	Endocellulase	*T. fusca*	9	16
1i4n	Indole-glycerol phosphate synthase	*T. maritima*	123	49
1thf	Imidazole-glycerol phosphate synthase	*T. maritima*	32	23
1igs	Indole-glycerol phosphate synthase	*S. solfataricus*	85	36
1dl3	Phosphorybosyl antranilate isomerase	*T. maritima*	22	17
1qo2	Ribonucleotide isomerase	*T. maritima*	101	42

For theozyme interactions 1 and 2, as described above, the catalytic geometry is fairly restricted, meaning that for all six degrees of freedom there are catalytically necessitated values. Therefore, the classic match algorithm was used to place these side chains. To increase the number of matches found, additional samples were done at small deviations from the ideal value. Theozyme interaction 3, featuring two degrees of freedom that can take on any value, is less clearly defined. Therefore the secondary matching algorithm was used to place the Lys, and during the matching process the torsion_B and torsion_AB parameters between candidate Lys rotamers and potential ligand placements were not evaluated. An overview over the matching setup is given in Table S1, and the results of the matching stage are in Table 1. In total, 372 unique matches were found.

### 3) Design

All unique matches were designed 10 times with the design protocol as described in the methods section. Specifically, the design shell was defined as all residues within a cut1 of 4 Å and cut2 6 Å of the ligand, and the repack shell as all residues within a cut3 of 10 Å and a cut4 of 12 Å. The catalytic residues as found by the matcher were considered to be part of the repack shell. This lead to design shells having on the order of 20 residues and repack shells on the order of 50 residues.

As a first step, all design shell residues were mutated to ALA, and a gradient-based optimization of the poly-A structure was done in order to optimize the catalytic interactions and ligand position (cst_opt stage).

The structures generated by the cst_opt stage were subjected to two rounds of design and minimization. During the design stage, interactions between the ligand and the protein were upweighted by a factor of 1.6, to favor the selection of sequences that make good protein-ligand interactions with the ligand over sequences that only make good protein-protein interactions. Further, to reduce the number of mutations that Rosetta introduces, at every design position the native residue identity was favored by a small bonus of 0.8 Rosetta energy units (REU). The purpose of this is to make sure that the native residue is kept at positions where there is no other residue that makes clearly better interactions, such as for exposed surface positions, hopefully avoiding inadvertent destabilization of the scaffold.

The structures thus generated were then repacked and minimized without the catalytic constraints, to ascertain the unbiased conformation of the designed sequence. Finally, to test whether the designed structures stay in the same conformation irrespective of the presence of the ligand, the ligand is removed and the structures repacked one last time. The complete protocol took on the order of 8 minutes per structure, and since a total of 3720 structures were generated, the complete CPU time for this stage was about 500 hours.

The resulting designs are then evaluated with respect to several factors as described in the methods section. The results are in Table 2. As a comparison, the values observed when repacking and minimizing the native 1ney enzyme are also listed.

**Table 2:** Design results.

Feature	av. value +− std.dev observed in 3270 designs	lowest/highest value observed in 3270 designs	av. value +− std.dev observed for repacking 1ney native 10 times	cutoff for selectingdesigns	# of designs passing cutoff
**ligand binding and catalytic site measures**					
total restraint score /REU	25.79+−31.95	0/303.21	3.50+−2.81	< 6.5	1255
restraint score catres1/REU	3.47+−6.01	0/149.45	0.76+−0.28	<1.2	1413
restraint score catres2/REU	3.89+−8.53	0/108.17	0.33+−0.20	<1.0	2136
restraint score catres 3/REU	5.53+−11.91	0/83.37	0.66+−1.57	<2.3	2696
ligand binding score/REU	−2.35+−1.34	−7.03/2.62	−9.77+−0.22	<−8.5	0
active site repack rmsd w/o ligand /Å	0.33+−0.21	0/1.46	0.06+−0.02	<0.5	3024
catres 1 repack rmsd w/o ligand Å	0.43+−0.76	0/4.43	0.12+−0.11	<0.5	2737
catres 2 repack rmsd w/o ligand Å	0.73+−0.79	0/4.56	0.0+−0.0	<0.5	1971
catres 3 repack rmsd w/o ligand Å	0.76+−0.94	0/5.28	0.06+−0.18	<0.5	2121
**scaffold integrity measures / differences to native**					
total score /REU	−91.35+−30.24	−158.97/186.32	n/a	<0.0	3705
# buried non H-bonded polar atoms	−2.10+−4.94	−19/19	n/a	<5	3413
# non-local contacts	6.16+−3.83	−10/19	n/a	>−2	3615
packstat [13]	−0.02+−0.04	−0.14/0.10	n/a	>−0.05	2565

### 4) Ranking and Selection

After the design stage, the question becomes which of the resulting 3720 designs to visually inspect and eventually select for expression. The philosophy applied here for selecting designs consists of two considerations: 1) for a design to be active, the ligand needs to have a good score (i.e. binding energy), the catalytic residues need to be in a competent conformation, and the active site needs to be preorganized; and 2) a designed protein must be folded, stable, soluble, and expressible in a standard *E.coli* production strain.

For consideration 1, since there is a natural enzyme known, this can be scored in Rosetta under the same conditions as the designs were generated and used as a benchmark. Designs are then only selected if they have comparable ligand-binding scores and comparable catalytic-constraint scores. In terms of preorganization, the RMSD of the catalytic residues between the final designed structure with a ligand and the structure that was repacked without the ligand has to be small. To enable selection of designs according to these criteria, the model of triose phosphate isomerase from S. cerevisia [11], was repacked and minimized in Rosetta, with the same cstfile as used in the designs.

Regarding consideration 2, it is important to note that the absolute Rosetta score does not necessarily correlate with properties such as protein stability, solubility, or a clearly defined fold. Yet, a designed protein should feature each of these. To judge the qualities of a certain design in this regard, one approach is to compare the designed protein to the scaffold that it originated from. Since the original scaffold was a well behaved protein, one can reasonably assume that a design based on it will also be, provided that not too many of the scaffold interactions and features have been corrupted. Here, designs were required to have comparable score, packing quality [13], contact order, and numbers of hydrogen bonds and buried polar atoms as the scaffolds they came from. Table 2 shows each feature that was used for selecting designs, together with the cutoffs used.

Of the 3720 designs, unfortunately none passed all the cutoffs, because none of the designs featured ligand binding scores comparable to the native. For the remaining selection parameters however, there were generally designs that had values comparable to the native active site or their respective scaffold. Not counting the ligand binding energy parameter, there were 43 designs that passed all cutoffs. The question then becomes if any of these 43 designs should be experimentally tested, or if matching in more scaffolds should be done to find matches that give rise to designs with better ligand binding scores. In the example design project presented here, only 5 starting scaffolds were used, whereas in most real-world design projects in our group, hundreds or thousands of scaffolds are considered. If one wanted to improve the binding score of the resulting designs, one possibility would be to include binding interactions with the ligand phosphate group (which is exquisitely bound in the 1ney native) in the theozyme, so that all matches would already feature better binding sites. Alternatively, the selected designs can be subjected to more thorough examination techniques such as MD simulations before experimental characterization is attempted [14].

## Discussion

The Rosetta3 enzyme design protocol as presented here constitutes a general method to create suggestions for protein catalysts, for any arbitrary reaction of interest. Though it has been shown to be capable of designing active enzymes in three cases [1], [2], [3], in each case the best designed proteins only had very modest activity, while many of the designs tested had no activity at all. Thus, while this protocol constitutes a powerful tool in the development of novel catalysts, success is by no means guaranteed. Several shortcomings and potentials for improvement exist, some of which have been showcased in this study. We consider three areas where we could improve the protocol.

First, to increase the quality of designs, it is beneficial to include as many interactions in the theozyme as possible, and concurrently run matching for as many scaffolds as possible. In the TIM example presented here, none of the designs showed sufficient ligand binding score, so for a new round of designs, it might be beneficial to include ligand binding interactions in the theozyme. However, the more complicated the theozyme becomes, the smaller the number of matches that are found; each additional geometric requirement made on the scaffold makes it less likely any particular scaffold will meet all the requirements. Incorporating backbone flexibility into the matching stage, possibly in a manner similar to the method developed by Havranek *et al.* [15], would likely increase the number of matches that can be found for complicated theozymes.

Second, the enzyme design protocol so far only considers one state of the reactant, or one snapshot of the reaction trajectory. This means that Rosetta will try to design a sequence that optimally stabilizes this state, while ignoring the other states that also occur along the reaction coordinate. For example, when designing a catalyst for a reaction featuring large spatial rearrangements of atoms, Rosetta might converge on a sequence that sterically clashes with one of the substrate- or product conformations. Natural enzymes have evolved to exquisitely compromise between stabilization requirements for every stage of the reaction trajectory [16]. To design efficient enzymes, it may be that all states of the reaction need to be modeled simultaneously, so that the designed sequence stabilizes the transition state more than any other, without destabilizing any other state too much. Developing a sequence selection algorithm that simultaneously takes a complete reaction trajectory into account is perhaps the biggest remaining challenge in computational enzyme design.

Third, ranking and selection of designs could be improved by the development of faster more thorough computational examination methods. Often, the catalytic side chains in the final designs will deviate from the idealized theozyme geometry. Further, the electrostatic potential created by the designed side chains that were not part of the theozyme also has an effect on the reaction's energy barrier, and thus the hypothetial stabilization achieved in a raw theozyme might be much less, depending on what scaffold this theozyme was placed in. Combined QM/MM hybrid approaches might be used to address this problem, but so far these methods are far too slow to be routinely employed for screening the hundreds or thousands of designs that Rosetta can suggest. Another factor that must be taken into account is the structural integrity of the designs. Even though the Rosetta designed sequence represents an energy minimum for the scaffold conformation, this does not mean that this sequence cannot fold into a different conformation. Local rearrangements of the backbone are not unlikely and have been reported for designed proteins. A possible method to examine designs for structural integrity is to run MD simulations [14], although this can quickly become prohibitively slow for large numbers of designs.

Despite the limitations listed above, from a purely practical standpoint, the Rosetta3 enzyme design protocol is still very useful. What distinguishes Rosetta's computational approach is that it is capable of generating catalytic activity from an inert scaffold, whereas most experimental methods, such as directed evolution approaches, rely on an existing catalytic activity as a starting point. Rosetta designed low-activity enzymes have been evolved to respectable catalysts [17], which shows that in combination with a high-throughput screening or selection strategy, Rosetta can facilitate the *de novo* creation of reasonable active enzymes.

## Supporting Information

Table S1 Theozyme geometries
(DOC)
