# Supplementary material for: De Novo Enzyme Design Using Rosetta3
Source: PLoS One. 2011 May 16;6(5):e19230. doi: 10.1371/journal.pone.0019230 (PMC3095599; doi:10.1371/journal.pone.0019230)
Supplement: Table S1 — Theozyme geometries (DOC) [file pone.0019230.s001.doc]

SI Table 1: Theozyme geometries

| parameter | ideal value +- tolerance | values sampled by matcher | num matcher samples | k_constraint_ |
| --- | --- | --- | --- | --- |
| **Interaction 1 Glu/Asp - DHAP** (total number of ligand placements per rotamer: 1*3*3*3*3*7= 567) |  |  |  |  |
| distanceAB/Å (E/D:Ocarboxy-DHAP:C1) | 3.06 +- 0.2 | 3.06 | 1 | 100 |
| angleA /º (E/D:Ocarboxy - DHAP:C1 - DHAP:C2) | 73.6 +- 10.0 | 63.6, 73.6, 83.6 | 3 | 80.0 |
| angleB /º (E/D:Cy/g - E/D:Ocarboxy - DHAP:C1) | 120.0 +- 15.0 | 105.0, 120.0, 135.0 | 3 | 80.0 |
| torsionA /º (E/D:Ocarboxy - DHAP:C1 - DHAP:C2 - DHAP:O2) | -101.2 +- 15.0 | -86.2, -101.2, -116.2 | 3 | 60.0 |
| torsionB /º( E/D:Cγ/β - E/D:Cδ/γ - E/D:Ocarboxy - DHAP:C1 ) | 180.0 +- 15.0 | 165.0, 180.0, -165.0 | 3 | 0.0 |
| torsionAB /º ( E/D:Cδ/γ - E/D:Ocarboxy - DHAP:C1- DHAP:C2 ) | 180.0 +- 90.0 | 90.0, 120.0, 150.0, 180.0, -150.0, -120.0, -90.0 | 7 | 0.0 |
| **Interaction 2 His - DHAP** (total number of ligand placements per rotamer: 1*3*3*3*3*10= 810) |  |  |  |  |
| distanceAB /Å (DHAP:O2 - His:Nε2) | 2.72 +- 0.20 | 2.72 | 1 | 100.0 |
| angleA /º (DHAP:C2 - DHAP:O2 - His:Nε2) | 111.2 +- 10.0 | 101.2, 111.2, 121.2 | 3 | 50.0 |
| angleB /º (DHAP:O2 - His:Nε2 - His:Cε1) | 120.3 +- 15.0 | 105.3, 120.3, 135.3 | 3 | 50.0 |
| torsionA /º (DHAP:C1 - DHAP:C2 - DHAP:O2 - His:Nε2) | 0.0 +- 10.0 | -10.0, 0.0, 10.0 | 3 | 50.0 |
| torsionB /º ( DHAP:O2 - His:Nε2 - His:Cε1 - His:Nδ1 ) | 180.0 +- 15.0 | 165.0, 180.0, -165.0 | 3 | 0.0 |
| torsionAB /º ( DHAP:C2 - DHAP:O2 - His:Nε2 - His:Cε1) | 0.0 +- 30.0;  180.0 +- 30.0 | -30.0, -15.0, 0.0, 15.0, 30.0 150.0, 165.0, 180.0, -165.0, -150.0 | 10 | 0.0 |
| **Interaction 3 Lys - DHAP**  (secondary match algorithm used) |  |  |  |  |
| distanceAB/Å (DHAP:O2 - Lys:Nζ ) | 2.90 +- 0.2 | n/a | n/a | 100.0 |
| angleA /º (DHAP:C2 -  (DHAP:O2 - Lys:Nζ ) | 109.3 +- 20.0 | n/a | n/a | 50.0 |
| angleB /º (DHAP:O2 - Lys:Nζ - Lys:Cε) | 109.8 +- 20.0 | n/a | n/a | 50.0 |
| torsionA /º (DHAP:C1 - DHAP:C2 - DHAP:O2 - Lys:Nζ ) | -100.0 +- 30.0 | n/a | n/a | 0.0 |
| torsionB /º (DHAP:O2 - Lys:Nζ - Lys:Cε - Lys:Cδ ) | any (0-360) | n/a | n/a | 0.0 |
| torsionAB /º (DHAP:C2 - DHAP:O2 - Lys:Nζ - Lys:Cε ) | any (0-360) | n/a | n/a | 0.0 |
